# Provision of Decentralized TB Care Services: A Detect–Treat–Prevent Strategy for Children and Adolescents Affected by TB

**DOI:** 10.3390/pathogens10121568

**Published:** 2021-12-01

**Authors:** Stella Zawedde-Muyanja, Anja Reuter, Marco A. Tovar, Hamidah Hussain, Aime Loando Mboyo, Anne K. Detjen, Courtney M. Yuen

**Affiliations:** 1 The Infectious Diseases Institute, College of Health Sciences, Makerere University, Kampala P.O. Box 22418, Uganda; 2 Médecins Sans Frontières, Cape Town 7784, South Africa; MSFOCB-Khayelitsha-Tbdoc@brussels.msf.org; 3 Socios En Salud Sucursal Perú, Lima 15001, Peru; mtovar_ses@pih.org; 4 Faculty of Health Sciences, Universidad Peruana de Ciencias Aplicadas, Lima 15067, Peru; 5 Interactive Research and Development Global, Singapore 238884, Singapore; hamidah.hussain@ird.global; 6 Elizabeth Glaser Pediatric AIDS Foundation, Kinshasa B.P. 1002030, Democratic Republic of the Congo; amloando@pedaids.org; 7 United Nations Children’s Fund, New York, NY 10017, USA; adetjen@unicef.org; 8 Department of Global Health and Social Medicine, Harvard Medical School, Boston, MA 02115, USA; Courtney_Yuen@hms.harvard.edu

**Keywords:** tuberculosis, decentralization, primary health care, patient-centered care, children, adolescents

## Abstract

In this review, we discuss considerations and successful models for providing decentralized diagnosis, treatment, and prevention services for children and adolescents. Key approaches to building decentralized capacity for childhood TB diagnosis in primary care facilities include provider training and increased access to child-focused diagnostic tools and techniques. Treatment of TB disease should be managed close to where patients live; pediatric formulations of both first- and second-line drugs should be widely available; and any hospitalization should be for as brief a period as medically indicated. TB preventive treatment for child and adolescent contacts must be greatly expanded, which will require home visits to identify contacts, building capacity to rule out TB, and adoption of shorter preventive regimens. Decentralization of TB services should involve the private sector, with collaborations outside the TB program in order to reach children and adolescents where they first enter the health care system. The impact of decentralization will be maximized if programs are family-centered and designed around responding to the needs of children and adolescents affected by TB, as well as their families.

## 1. Introduction

Approximately one out of every six people who develop TB every year is a child or adolescent under 20 years old [1,2]. A significant proportion do not receive appropriate treatment for TB either because they are not diagnosed; are diagnosed but not started on TB treatment; or are treated inappropriately. The COVID-19 pandemic has resulted in challenges to accessing TB services that have compounded this problem, resulting in a 24% decrease in notifications in the 0–14 age group and an 11% reduction in child contacts receiving TB preventive treatment (TPT) in 2020 compared to 2019 [1]. Children and adolescents have distinct needs with respect to TB diagnosis, treatment, and prevention services compared to adults [3,4]. Younger children are more likely than adults to have paucibacillary or disseminated disease, so they require diagnostic services that do not rely on sputum testing [3]. Children and adolescents exposed to TB bacilli are at elevated risk for developing TB disease once infected and, therefore, are a priority population for preventive treatment [5].

In order to provide high-quality TB care for children and adolescents, services catering to their needs must be available at the points where they enter the health system. Children and adolescents affected by TB require access to radiography, gastric lavage, pediatric TB specialists, and youth-friendly clinics. However, these services are often concentrated in referral-level hospitals or specialized facilities. By contrast, children and adolescents generally access the health system through outpatient or maternal-child health clinics at primary care facilities or private general practitioners [4]. This mismatch between the location of services and the location of care-seeking leads to losses from care and delays in TB diagnosis, treatment, and prevention.

In order to avoid missed or delayed opportunities for TB care, it is necessary to decentralize child- and adolescent-appropriate TB services by making these services available at the points of the health system where care is first sought. Health care workers at these care points must possess the clinical training to elicit a relevant patient history; conduct a thorough physical exam; order the appropriate tests to diagnose TB; and manage treatment of disease and preventive treatment. In this article, we review considerations and successful models for providing decentralized diagnosis, treatment, and prevention services for children and adolescents.

## 2. Decentralizing Diagnostic Services

The diagnosis of TB in children can be challenging due to symptomatic overlap with other common childhood diseases such as pneumonia, viral respiratory tract infections, and asthma. Younger children often having difficulties expectorating sputum, and the paucibacillary nature of childhood TB infections makes bacteriological confirmation less likely [3]. A key approach to building decentralized capacity for childhood TB diagnosis is training and continued onsite mentorship for primary care providers to build and maintain the clinical competencies required to diagnose child and adolescent TB [6]. Providers must be trained to screen for TB when children enter the health system; take a detailed history including asking about TB exposure (possibly using an exposure score [7]); and elicit clinical signs consistent with active TB. In TB-endemic settings, primary care providers must be trained to consider TB as a potential diagnosis when faced with nonspecific symptoms (e.g., weight loss or failure to thrive, defined as persistent weight below the third percentile for age [8]), particularly in children with a history of TB exposure. Although most of this training currently takes place among in-service health care workers, efforts must be made to incorporate these clinical competencies into pre-service training, particularly of non-physician clinicians who are the main providers of basic clinical services in primary care settings in countries with high TB burdens [9].

Child-focused diagnostic tools must also be made available at the primary care level. Capacity for radiography must be increased in primary care facilities, with physicians trained in radiographic diagnosis of pediatric TB or with the use of computer-aided detection software—where possible—to triage children or adolescents with abnormal chest radiographs for further evaluation [10]. Finally, providers should be trained to collect stool samples [11] and induced sputum samples for the bacteriological confirmation of TB. Although bacterial confirmation is challenging in children, a bacteriological sample is invaluable for diagnosing drug resistance.

In Uganda, the DETECT Child TB (Decentralize TB services and Engage Communities to Transform lives of Children with TB) trained primary care providers to screen children for TB, perform detailed clinical exams, and perform child-focused specimen collection techniques (e.g., gastric lavage) [12]. The project also strengthened laboratory functionality at the primary level and implemented community-based contact investigation. Initial implementation in two districts resulted in a two-fold increase in the number of children diagnosed with TB, the majority of whom were clinically diagnosed. Countrywide roll-out of decentralized care for child TB over the past five years has resulted in a sustained increase in TB diagnosis among children. By 2019, children and adolescents younger than 15 years made up 13% of all TB cases notified countrywide compared to 7% prior to the implementation of decentralized care.

Older children and adolescents have higher rates of bacteriological confirmation of active TB but face unique challenges in accessing TB services [4]. These include stigma due to fear of exclusion from school and other social gatherings and peer pressure. In order to improve TB diagnosis among adolescents, it is necessary to integrate TB services into ongoing youth-focused interventions and adolescent-friendly youth services at primary care facilities. This approach has been used in HIV care, resulting in increased ART uptake, improved retention in care, and comparable mortality to centralized care provision [13].

## 3. Decentralizing Treatment of TB Disease

Children and adolescents diagnosed with both drug-sensitive and drug-resistant TB should be managed in the community and in the health facilities nearest to them. Community outpatient-based treatment is associated with improved treatment outcomes, faster time to treatment initiation [14,15], and reduced pre-treatment loss to follow-up [16]. Community-based care is also substantially more cost-effective than centralized models of treatment [17]. While a period of hospitalization may be required for patients who are acutely unwell and require stabilization or who are suffering from a TB-related complication such as TB meningitis, this should be for as brief a period as medically indicated. Moreover, when children and adolescents are discharged from the hospital to continue treatment in more decentralized outpatient settings, it is important to establish robust linkage mechanisms to avoid losses from care [18].

In many countries, treatment for drug-resistant TB is centralized at specialist hospitals. Even when treatment is ambulatory, families are unable to travel away from the location of the hospital for the long duration of treatment, thereby disrupting their social lives [19]. In instances where children and adolescents are hospitalized for prolonged periods away from their families, there can be catastrophic financial impacts for families [20], as well as psychosocial and health repercussions on the child or adolescent [21,22]. Disruption of parent-child bonding and cessation of breast feeding for young infants; missing out on school and social isolation in older children and adolescents can all have long-lasting impacts.

Recent advances in treatment with new and repurposed drugs for drug-resistant TB have increased the feasibility of managing drug-resistant TB for children and adolescents in decentralized facilities, without hospitalization. New all-oral regimens are simpler, safer and more effective, and they eliminate the need for administering injectable agents and the associated ototoxicity monitoring. With sufficient training and clinical support, primary care providers can initiate drug-resistant TB treatment, monitor and manage adverse events, and achieve excellent treatment outcomes [23]. First- and second-line TB drugs need to be made readily available at the primary care level, and access to pediatric formulations of these medications is an urgent priority to simplify treatment both for caregivers, children, and health workers.

## 4. Decentralizing Preventive Services for Child and Adolescent Contacts

Children and adolescents exposed to both drug-sensitive and drug-resistant TB bacilli should be given TPT. Situating TPT services closer to families affected by TB is important for bringing exposed children and adolescents into care. Community health workers can conduct the initial step of contact identification and screening via home visits [12,24]. These visits are an opportunity not only to identify contacts who may require attention, but to provide TB education and emotional support, which can help promote the uptake of preventive services. Where resources are limited, home visits can be targeted to households where the risk of incident TB is highest, which includes those where either the index patient or contacts are children and adolescents [25]. Providing socioeconomic support to TB-affected families to offset the costs associated with accessing preventive services can also increase the number of contacts accessing care [26].

Primary care facilities must have sufficient diagnostic capacity to rule out TB and initiate TPT. Where access to radiography is limited, TB can be ruled out using clinical algorithms, which can be applied by a nurse or clinical officer. As a negative symptom screen has high concordance with non-TB-suggestive radiography [27], TPT can be initiated immediately for asymptomatic child and adolescent contacts, while those with symptoms can be monitored or prioritized for further evaluation before deciding whether to initiate TPT or treatment for active TB. Where decentralized TB infection testing is not feasible, initiating TPT for all child and adolescent contacts once active TB disease has been ruled out is a reasonable policy given their high risk of disease progression and the low risk of serious side effects [28].

Short TPT regimens with self-administration and support can improve the feasibility of managing increasing numbers of child and adolescent contacts in decentralized settings. A 3-month weekly (12-dose) regimen as well as a 1-month (30-dose) regimen of isoniazid and rifapentine have both proven feasible for delivery to child and adolescent contacts in a high-burden setting, with improved completion compared to 6 months of daily isoniazid [29,30]. Fluoroquinolone-based preventive regimens for drug-resistant TB, while longer, have also been used programmatically in high-burden settings with good completion [31,32].

## 5. Engaging the Private Sector

In many countries, substantial proportions of people with TB, including children and adolescents, first seek care from the private sector [33,34]. However, in many settings, there is a lack of knowledge of TB among private health providers; low TB diagnostic capacity in the private sector; and a lack of mechanisms for notification of patients diagnosed in the private sector to national TB programs (NTPs) [35]. Several successful models for engagement of the private sector to improve child and adolescent TB services have been demonstrated. For example, training in TB diagnosis and management can be offered to private providers, with a form of certification offered to provide legitimacy and make training more desirable [36,37]. Establishing in-person or digital patient referrals [38,39] or sputum transportation networks [40] can improve diagnosis for children and adolescents accessing private general practitioners or pharmacies, especially benefitting children with atypical presentations of TB. Treatment can be provided in the private sector, with free medications provided by the NTP [36,37]. Harmonization of reporting mechanisms with NTPs is important for monitoring service provision in the private sector, as is already performed in the public sector [41]. These approaches have been shown to increase private practitioners’ engagement in TB care provision, leading to dramatic increases in the number of children and adolescents started on TB treatment [38,40], and good outcomes for children and adolescents treated in the private sector [36]. Moreover, public–private partnership between NTPs and the private sector can reduce out-of-pocket costs for patients and increase the cost-effectiveness of service delivery [42,43].

## 6. Integrating TB Care with Other Services for Children and Adolescents

National TB strategic plans increasingly recognize the importance of strengthening the integration of TB into child and adolescent health programs. However, TB is not systematically included in key policy frameworks, strategies, and operational tools to reduce child mortality, such as Integrated Management of Neonatal and Childhood Illnesses (IMNCI), or the management of acute malnutrition [44,45]. Providers of child and adolescent health services are not necessarily trained in basic TB prevention, diagnosis, and care; TB services are often managed and delivered separately by different providers [46]. Considerations around successful integration include: the selection of high yield service delivery points; human resource requirements (training as well as ongoing mentoring and supervision); referral pathways; and monitoring. It is important for TB care to be included when operational plans and child/adolescent health strategies are developed for primary care settings as well as for community health worker programs.

Efforts in high-burden settings to integrate TB screening into primary care settings such as outpatient clinics and IMNCI clinics have been shown to increase the referral of children at risk of TB, as well TB case detection [12,47]. The ongoing multi-country Catalyzing Pediatric TB Innovations (CaPTB) project has undertaken the systematic integration of TB screening and strengthening of decentralized diagnostic capacity at different service delivery entry points, including outpatient, inpatient, well-child, nutrition, and HIV clinics. This project showed a 1.6-fold increase in the number of children diagnosed with TB, with the highest yield in services providing care for sick children. The intervention was well accepted by health care workers and facility personnel, but additional human resources were required to support TB screening. Several countries shifted tasks to community health workers, which was feasible and well accepted.

Other opportunities for the integration of TB screening and diagnosis exist in the context of programs that provide services for children and adolescents with risk factors for TB. Integrating TB screening into malnutrition programs could promote earlier diagnosis, as malnutrition is a major cause or co-morbidity in children with TB [44]. Integrating TB and HIV treatment for children and adolescents living with TB can reduce the number of clinic visits required and improve the coordination of counseling and support for both conditions [48]. Finally, collaborations with service providers outside the health care system who work with high-risk children and adolescents could help to reach individuals who might not otherwise access the health care system [49].

## 7. Recommendations

Primary health care aims to ensure universal coverage of basic health care through quality and integrated service delivery, multisector coordination, as well as the engagement and empowerment of communities. Offering TB services as part of the primary health care package delivered at frontline public or private health facilities, or in the community, can help improve diagnosis, treatment, and prevention. We can learn from the success of programs that have strengthened decentralized and integrated TB services for children and adolescents affected by TB. Key strategies are described in Table 1.

## 8. Conclusions

In order to close the widening gap in access to TB health care services caused by the COVID-19 pandemic, we must increase the reach and effectiveness of TB diagnostic, treatment and preventive services. Decentralization of TB care provision is an important strategy to achieve this. Effective decentralization requires training of existing staff, investment in new staff cadres including community health care workers, improving access to medications and diagnostics and integration of TB services into existing primary care services. Finally, the impact of decentralization will be maximized if programs are designed around responding to the needs of children and adolescents affected by TB as well as their families.

## Figures and Tables

**Table 1:** Key strategies for decentralizing TB care for children and adolescents.

Strategy	Considerations
Expand human resources for TB services	When a new service such as TB screening or preventive treatment is introduced into a health facility, a commensurate increase in human resources is required. Task-shifting and the employment of community health workers can help reduce costs associated with increased staffing.
Provide training, support, and mentorship for primary care providers	Primary care providers often lack knowledge around TB diagnosis, treatment, and prevention for children and adolescents, particularly drug-resistant TB. These topics should be incorporated into pre-service as well as in-service training, and training programs should reach providers in both the public and private sectors. In addition to didactic training, ongoing support and mentorship from experienced providers or specialists is important. This can be accomplished through onsite visits or remotely through one-on-one virtual mentorship, or through digital platforms (e.g., Project ECHO) that enable collaborative case reviews among groups.
Expand access to new diagnostic modalities and treatments	New diagnostic modalities that enable quick and accurate detection of TB should be made available in decentralized health facilities (e.g., computer-aided detection software for chest X-rays, rapid molecular testing, lateral flow urine lipoarabinomannan assay). In addition, oral pediatric formulations of drugs for both treatments and prevention of both drug-susceptible and drug-resistant TB should be made widely available to enable healthcare workers to manage children and adolescents as close to their homes as possible.
Expand TB preventive treatment (TPT)	While diagnosis, treatment, and prevention are all critical, the largest gap currently exists for TPT, particularly for children and adolescents over 5 years old. In some countries, national guidelines must be changed to indicate TPT for these older age groups, as well as to recommend shorter regimens and TPT for drug-resistant TB contacts. Primary care providers must be trained in pragmatic algorithms for initiating TPT, and sufficient human resources must be provided to support the increased patient load.
Provide family-centered care	Services should be designed to minimize the burden placed upon families affected by TB. A family-friendly clinic would schedule appointments together for family members receiving treatment and preventive therapy, and provide health education, age-appropriate adherence support, and socioeconomic support. Ideally, the same providers would manage both adults and children in order to make visits more efficient and allow for holistic consideration of the family’s needs.
Collaborate outside the TB program	TB services should be provided where children and adolescents access the health care system, including outpatient clinics, IMCNI clinics, adolescent/youth friendly clinics, and private general practitioners. Providing TB services at these points will require collaboration between NTPs and relevant stakeholders to integrate strategies and services.
Collect and use age-disaggregated data to monitor programs	Continuous monitoring and evaluation are required to assess the impact of decentralization and ensure that the quality of TB services remains high. Programs should routinely use their data to assess care cascades for diagnosis, treatment, and prevention, thereby embedding operational research to improve service delivery where possible. Collection of age-disaggregated data (e.g., 0–4, 5–9, 10–14, 15–19 years) will promote a deeper understanding of the disparities in TB care access within child and adolescent age groups.
